# Can continuous glucose monitoring-derived glycemic variability predict retinal neurodegeneration earlier than HbA1c in patients without clinically apparent retinopathy?

**DOI:** 10.3389/fendo.2026.1925994

**Published:** 2026-08-25

**Authors:** Yixiang Su, Guang Han, Qinghua Su

**Affiliations:** 1 Changchun University of Chinese Medicine, Changchun, Jilin, China; 2 Department of Ophthalmology, Pingyang Branch, Changchun Hospital of Traditional Chinese Medicine, Changchun, Jilin, China

**Keywords:** continuous glucose monitoring, diabetic retinopathy, early warning, glycated hemoglobin, glycemic variability, retinal neurodegeneration, time in range

## Abstract

Early screening for diabetic retinopathy (DR) has long relied on the detection of fundus vascular lesions. However, accumulating evidence indicates that retinal neurodegeneration may occur before overt microvascular abnormalities become clinically visible. At the stage of no clinically apparent diabetic retinopathy (NDR), glycated hemoglobin A1c (HbA1c), by virtue of its averaging nature, is unable to adequately reflect the acute metabolic stress imposed by short-term glycemic variability (GV) on the retinal neurovascular unit (NVU). The increasing use of continuous glucose monitoring (CGM) has made dynamic glycemic metrics such as time in range (TIR) and coefficient of variation (CV) clinically accessible. This review summarizes the clinical and mechanistic evidence supporting CGM-derived GV metrics in the association with early retinal injury in patients with NDR. Mechanistic studies indicate that fluctuating hyperglycemia induces mitochondrial oxidative stress more readily than sustained hyperglycemia and can activate Müller cell- and microglia-mediated neuroinflammation while disrupting tight junctions within the blood-retinal barrier. Clinical studies further show that reduced TIR and elevated CV are associated with early thinning of the retinal nerve fiber layer and reduced microvascular density, and some of these associations remain significant after adjustment for HbA1c. On this basis, we propose a dual-dimensional risk assessment strategy combining HbA1c with TIR/CV and suggest that patients with NDR who exhibit marked glycemic variability on CGM should undergo intensified monitoring. Integrating dynamic CGM metrics with optical coherence tomography (OCT) and OCT angiography (OCTA) may enable risk stratification during the NDR stage and shift the intervention window for DR forward to a critical phase for neuroprotection.

## 1 Introduction

Diabetic retinopathy (DR) is one of the most common and most destructive microvascular complications of diabetes and has long been regarded as a typical consequence of chronic hyperglycemia-induced vascular endothelial injury and blood-retinal barrier (BRB) breakdown (1). Globally, approximately one-third of people with diabetes have some degree of retinal involvement, and DR remains the leading cause of vision loss among working-age adults (2). However, with the widespread adoption of high-resolution retinal imaging modalities such as optical coherence tomography (OCT) and OCT angiography (OCTA), together with advances in mechanistic studies of the retinal neurovascular unit (NVU), it has become increasingly clear that retinal injury does not necessarily begin with clinically visible microaneurysms or hemorrhages (3). In patients with diabetes who have not yet developed clinically apparent diabetic retinopathy, retinal ganglion cell (RGC) apoptosis, retinal nerve fiber layer (RNFL) thinning, and subclinical microcirculatory abnormalities have already been documented in multiple studies (4, 5). This conceptual shift, namely that neurodegeneration may precede vasculopathy, suggests that conventional screening strategies centered on late vascular lesions may miss the critical window for early intervention.

In the search for drivers of early retinal injury, substantial limitations have emerged in conventional glycemic metrics. Glycated hemoglobin A1c (HbA1c), the gold standard for long-term glycemic assessment, primarily reflects mean blood glucose over the preceding two to three months (6), yet it cannot adequately characterize short-term glycemic variability (GV), abrupt alternation between hyperglycemia and hypoglycemia, or marked circadian fluctuations in glucose levels (7–9). Increasing evidence suggests that pronounced glucose fluctuations induce oxidative stress, inflammatory responses, and endothelial apoptosis more readily than sustained hyperglycemia alone, thereby causing early injury to vulnerable retinal neurons (10–13). By contrast, the expansion of continuous glucose monitoring (CGM) has made it possible to obtain high-resolution dynamic glycemic data. CGM-derived metrics of GV, particularly time in range (TIR) and coefficient of variation (CV), can more sensitively capture metabolic instability and provide an additional dimension for identifying patients who meet HbA1c targets but remain at elevated risk for complications (7, 14–16).

**Figure 1:**
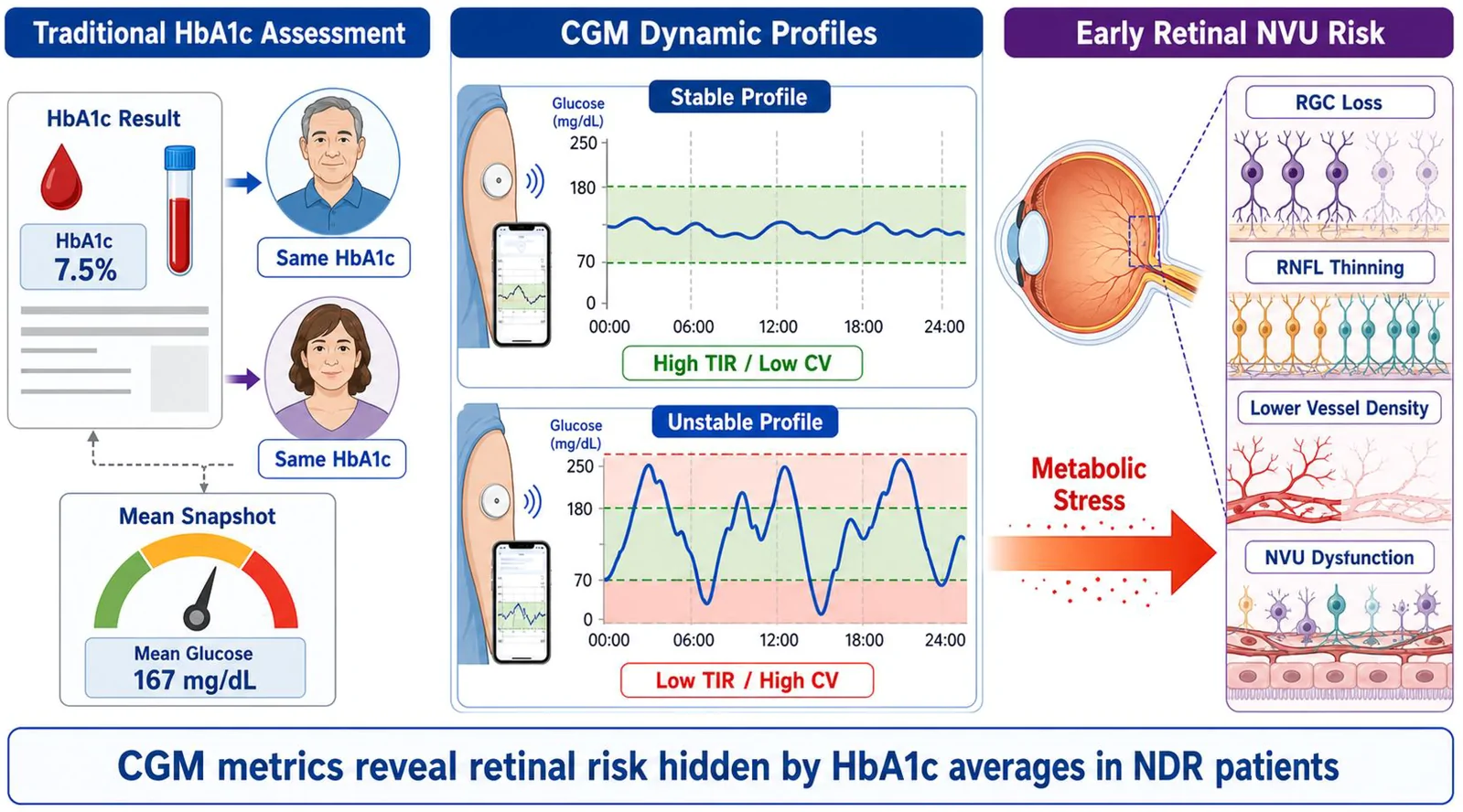
The HbA1c “masking effect of the mean” and CGM-based dynamic risk identification. Patients with the same HbA1c level may exhibit markedly different intraday glucose trajectories. CGM can capture dynamic metabolic features such as reduced TIR, elevated CV, and peak-to-trough fluctuations, thereby identifying NDR patients whose HbA1c is at target but who remain under heightened metabolic stress, and suggesting that they may already have early retinal neurovascular unit injury. TIR, Time in Range; CV, Coefficient of Variation; NVU, Neurovascular Unit; NDR, No Diabetic Retinopathy; RNFL, Retinal Nerve Fiber Layer; RGC, Retinal Ganglion Cell.

Against this background, whether CGM-derived GV metrics can signal retinal neural injury earlier and more sensitively than HbA1c has become a key scientific question in the early prevention and management of diabetic complications. Focusing on patients with no clinically apparent diabetic retinopathy, this review summarizes and compares the clinical and mechanistic evidence regarding CGM-derived GV and HbA1c in the association with early retinal neurovascular injury, and it discusses the potential molecular mechanisms by which glycemic fluctuations drive early retinal neurodegeneration. Unlike existing reviews that have focused largely on vascular outcomes in established DR or on microvascular complications in general, the present review is distinct in three respects: it focuses specifically on the NDR stage rather than established DR; it takes retinal neurodegeneration (RNFL thinning, ganglion cell layer thinning, neural dysfunction) as the primary outcome rather than general microvascular lesions; and it systematically links CGM-derived metrics with subclinical retinal structural changes detectable by OCT/OCTA, elucidating the mechanistic logic underlying this connection. The aim is to provide a basis for reexamining early screening strategies for diabetic eye disease and to open new directions for risk stratification and individualized intervention based on dynamic glycemic patterns. The core scientific question and its logic chain are illustrated in **Figure 1**.

## 2 Methods

This is a narrative review. A systematic literature search was conducted using PubMed, Web of Science, and the Cochrane Library, covering the period from database inception to June 2026, with language restricted to English. The search strategy combined the following Medical Subject Headings and free-text terms: (“continuous glucose monitoring” OR “CGM” OR “glycemic variability” OR “time in range” OR “TIR” OR “coefficient of variation” OR “CV”) AND (“diabetic retinopathy” OR “retinal neurodegeneration” OR “retinal nerve fiber layer” OR “neurovascular unit” OR “OCT” OR “OCTA”) AND (“HbA1c” OR “glycated hemoglobin”). Inclusion criteria were: (1) studies involving patients with no clinically apparent retinopathy or early DR; (2) use of CGM-derived glycemic variability metrics (TIR, CV, SD, MAGE, TITR, etc.); (3) assessment of retinal structural (OCT/OCTA-measured RNFL, GC-IPL thickness, capillary density, FAZ, etc.) or functional (ERG, etc.) outcomes; and (4) experimental studies investigating mechanisms linking glycemic variability to retinal injury. Exclusion criteria were: (1) studies focusing exclusively on proliferative DR or advanced diabetic macular edema; (2) studies without CGM or glycemic variability assessment; and (3) conference abstracts, editorials, and non-peer-reviewed literature. In addition, the reference lists of included articles were manually searched to identify any potentially relevant studies that may have been missed. As this is a narrative review, no formal quality assessment or risk-of-bias evaluation was performed.

## 3 Traditional metrics such as HbA1c cannot effectively signal early retinal injury

### 3.1 The HbA1c “masking effect of the mean” obscures the true risk of glycemic variability

HbA1c is an irreversible product formed by nonenzymatic glycation of hemoglobin with glucose. Its rate of formation is proportional to the mean glucose concentration over the life span of erythrocytes, and it has therefore been widely used as the gold-standard indicator of overall glycemic control during the preceding 8–12 weeks (6). However, the biological nature of HbA1c means that it can provide only an averaged snapshot over a defined period and cannot distinguish among different dynamic glucose patterns. A classic study showed that two patients with diabetes with the same HbA1c value could nevertheless have markedly different complication risks: individuals with greater biological variability, measured by the hemoglobin glycation index, had approximately a threefold higher risk of retinopathy and a sixfold higher risk of nephropathy than those with lower variability (17). This “masking effect of the mean,” as illustrated in **Figure 1**, whereby patients with the same HbA1c level may exhibit markedly different intraday glucose trajectories and whereby CGM captures reduced TIR and elevated CV to reveal metabolic instability hidden beneath the HbA1c average, implies that patients with identical HbA1c levels may experience substantially different degrees of metabolic stress at the level of target organs. Ceriello and colleagues further demonstrated that acute glucose fluctuations impair endothelial function more severely than sustained hyperglycemia of comparable mean intensity, indicating that the dynamic fluctuation of glucose itself is an independent driver of vascular injury (18). Therefore, reliance on HbA1c alone may misclassify patients with marked glycemic fluctuations but acceptable average glucose levels as being at low risk, thereby delaying early intervention.

### 3.2 Early microvascular and neural injury may continue to progress despite target HbA1c levels

Large-scale clinical trials have established that maintaining HbA1c below 7.0% significantly reduces the incidence and progression of DR (19). Nevertheless, early retinal injury is not fully prevented in patients whose HbA1c is at target. Using swept-source OCT (SS-OCT) in patients with type 2 diabetes (T2D) who had mild DR or no DR, da Silva et al. found that the subgroup with diabetic kidney disease had significantly reduced macular retinal thickness and capillary density compared with controls, whereas no statistically significant difference was observed in those without clear kidney involvement (3). Pedersen et al. reported in a T2D cohort that patients with mild cognitive impairment (MCI) had thinner macular nerve fiber and ganglion cell layers than those without MCI, suggesting that retinal neural thinning may represent a peripheral manifestation of systemic neurodegeneration (4). Analysis of CGM data from patients with latent autoimmune diabetes in adults (LADA) and T2D by Lu et al. showed that glucose variability, measured by standard deviation, was independently associated with DR in T2D and remained significant after adjustment for HbA1c (20). Picconi et al. further showed that glucose variability was an independent predictor of early retinal neural structural changes in type 1 diabetes (T1D), and that its effect was independent of HbA1c (21). Collectively, these findings indicate that HbA1c lacks sufficient sensitivity for the detection of cellular-level neural injury, whereas CGM-derived dynamic metrics may capture early signals of tissue damage before changes in HbA1c become evident.

### 3.3 Clinical practice urgently needs dynamic risk indicators that reflect real-time metabolic stress

Current screening systems for diabetic eye disease rely primarily on color fundus photography or fluorescein angiography to detect microaneurysms, hemorrhages, and exudates, all of which represent relatively late structural vascular lesions (1). Their fundamental limitation is that by the time vascular lesions become detectable, retinal neurodegeneration and NVU dysfunction have often already progressed substantially, and the window for neuroprotection may have been missed (5). Several guidelines have suggested incorporating OCT-based assessment of neural layer thickness into early DR screening (3), but in the absence of effective risk stratification tools, the cost-effectiveness of performing high-resolution OCT screening in all patients with NDR remains uncertain. Accordingly, clinical practice requires a dynamic biomarker capable of reflecting real-time metabolic stress and signaling tissue injury before vascular lesions become clinically visible. An ideal indicator should capture the dynamic features of glycemic fluctuations, provide early detection of target-organ metabolic stress before HbA1c changes, and be noninvasive, repeatable, and clinically accessible. CGM-derived GV metrics meet these requirements and offer a practical means of filling the information gap between average glycemic assessment and early tissue injury identification (7, 14).

## 4 CGM-derived glycemic variability metrics can precisely characterize metabolic instability

### 4.1 Continuous glucose monitoring has enabled a shift from “point sampling” to around-the-clock dynamic assessment

CGM systems use subcutaneous glucose sensors to measure interstitial glucose concentrations continuously at intervals of 1–5 minutes, thereby generating a 24-hour glucose profile and overcoming the limitation of conventional finger-stick testing, which provides only discrete snapshots (22). In a study of reference CGM values in healthy nondiabetic individuals, Shah et al. found that the median time spent within 3.9-7.8 mmol/L, defined as the time in tight range for healthy physiology, was approximately 96%, whereas the time spent below 3.9 mmol/L was approximately 1.1% (about 15 minutes per day), thereby providing a normal baseline against which abnormal glycemic fluctuations in diabetes can be judged (22). CGM can sensitively capture occult glycemic abnormalities that are difficult to identify using conventional methods, including asymptomatic nocturnal hypoglycemia, postprandial hyperglycemic peaks, and morning glucose drift associated with the dawn phenomenon (7). These hidden events cannot be captured by HbA1c yet constitute major sources of metabolic stress capable of inducing endothelial injury and neural apoptosis.

### 4.2 Classification of core glycemic variability metrics and their pathophysiological significance

International consensus broadly classifies CGM-derived metrics into amplitude-based and time-based indices (14, 16). The definitions and thresholds of the core metrics are summarized in **Table 1**.

**Table 1:** Definitions and thresholds of core CGM-derived glycemic variability metrics.

Metric	Abbreviation	Definition	Threshold
Time in Range	TIR	Percentage of time glucose is within 3.9-10.0 mmol/L	>70% (14)
Time in Tight Range	TITR	Percentage of time glucose is within 3.9-7.8 mmol/L	No unified threshold
Time Above Range	TAR	Percentage of time glucose >10.0 mmol/L	<25% (14)
Time Below Range	TBR	Percentage of time glucose <3.9 mmol/L	<4% (14)
Coefficient of Variation	CV	Ratio of glucose SD to mean glucose	≤36% (14)
Standard Deviation	SD	Standard deviation of glucose values	No unified threshold
Mean Amplitude of Glycemic Excursion	MAGE	Average amplitude of “effective” excursions (>1 SD)	No unified threshold

TIR, Time in Range; TITR, Time in Tight Range; TAR, Time Above Range; TBR, Time Below Range; CV, Coefficient of Variation; SD, Standard Deviation; MAGE, Mean Amplitude of Glycemic Excursion. Thresholds are based on the 2019 international consensus by Battelino et al. (14). In the context of early retinal neural injury, TIR is suitable for assessing the cumulative burden of hyperglycemic exposure, whereas CV is suitable for assessing the acute impact intensity of glycemic fluctuations on neurons and micro vessels. Their combined use may provide complementary risk information.

Amplitude-based indices include standard deviation (SD), coefficient of variation (CV), and mean amplitude of glycemic excursions (MAGE). CV, defined as SD divided by mean glucose and expressed as a percentage, is a standardized parameter for measuring glucose dispersion, and international consensus regards CV ≤36% as an acceptable threshold (14, 23). MAGE reflects the average amplitude of “effective” glucose excursions exceeding 1 SD and is considered particularly specific for capturing marked intraday fluctuations. Time-based indices include TIR, time above range (TAR), and time below range (TBR). TIR is currently the most widely discussed CGM parameter in clinical research. An analysis based on a large T1D cohort from the Diabetes Control and Complications Trial (DCCT) showed that each 10% decrease in TIR was associated with an approximately 64% increase in the risk of retinopathy progression and an approximately 40% increase in the risk of microalbuminuria (24). From a pathophysiological perspective, amplitude-based metrics such as CV and MAGE mainly reflect the severity of peak-to-trough glucose differences, and their pathogenicity is closely linked to intermittent hyperglycemia-induced acute mitochondrial superoxide generation (10, 25). Time-based metrics such as TIR and TAR reflect the cumulative proportion of time spent within metabolically safe or harmful hyperglycemic ranges, and their injurious logic lies in the accumulation of advanced glycation end-products (AGEs) and sustained activation of the polyol pathway under persistent hyperglycemia (7, 26). Together, these two categories of metrics characterize the pathological significance of glycemic fluctuations from different dimensions and jointly form a framework for evaluating metabolic stability (27, 28).

### 4.3 Glycemic variability has been recognized as an independent risk factor for microvascular complications

**Figure 2:**
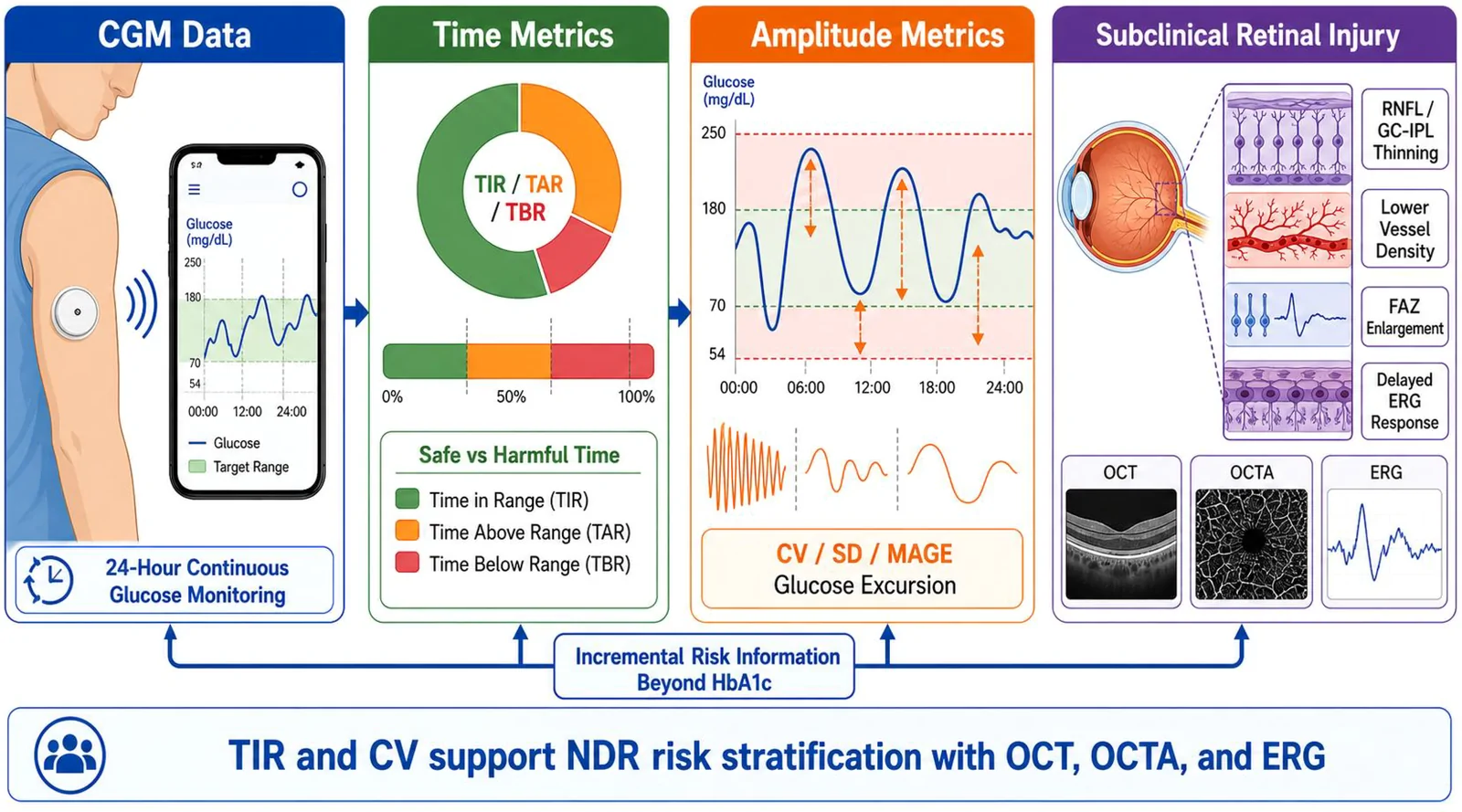
CGM-derived metrics and subclinical phenotypes of early retinal injury. CGM metrics characterize metabolic instability from both time-based and amplitude-based dimensions. TIR, TAR, and TBR reflect the cumulative time spent within target, hyperglycemic, or hypoglycemic ranges, whereas CV, SD, and MAGE reflect the degree of glucose dispersion and the intensity of peak-to-trough excursions. These dynamic metrics can be integrated with OCT-, OCTA-, and electroretinography-detected changes such as RNFL or ganglion cell-inner plexiform layer thinning, reduced capillary density, enlargement of the foveal avascular zone, and neural dysfunction to support early risk identification during the NDR stage.

Lu et al. were among the first to show, using CGM, that glucose variability measured by SD was associated with DR risk independently of HbA1c, suggesting that glucose variability itself is an independent risk factor for DR (20). In a cross-sectional study of 808 patients with T1D, De Meulemeester et al. confirmed that TIR and time in tight range (TITR, 3.9-7.8 mmol/L) were negatively associated with multiple microvascular complications, including DR, diabetic kidney disease, and diabetic peripheral neuropathy, and that some of these associations remained independent after adjustment for HbA1c (29). Using the virtual CGM (vCGM) dataset derived from the DCCT, Lobo et al. found a dose-response relationship between TIR/TITR and retinopathy progression endpoints, with associations comparable in strength to HbA1c (30). A review by Anagnostopoulou et al. integrated the evidence linking CGM parameters to microvascular outcomes and concluded that combined assessment of TIR, GV, and TITR may compensate for the information blind spots of HbA1c in predicting microvascular complication risk (31). Taken together, glycemic variability, whether quantified through amplitude-based or time-based metrics, has become an important risk factor for diabetic microvascular complications, thereby providing an evidence base for incorporating it into systems for the early identification of retinal neural injury. The relationship between CGM metrics and subclinical retinal injury phenotypes is illustrated in **Figure 2**.

## 5 The retinal neurovascular unit already exhibits subclinical injury before clinically visible vascular disease appears

### 5.1 The conceptual shift that “neurodegeneration precedes vasculopathy” is supported by substantial evidence

DR has long been defined as a disease centered on microvascular injury, with retinal neurodegeneration traditionally regarded as a downstream consequence secondary to vascular damage. Research over the past two decades has altered this view. Antonetti et al. proposed that retinal neuronal apoptosis and synaptic density reduction occur early in DR, even before vascular lesions become fully manifest, and defined this process as “diabetic retinal neurodegeneration,” a pathological process with clinical relevance independent of microvascular damage (5, 32, 33). The 4-year prospective cohort study by Sohn et al. provided strong evidence for this concept: among 45 patients with diabetes who had no DR or only minimal DR, the RNFL and ganglion cell/inner plexiform layers thinned progressively at a rate of approximately 0.25-0.29 μm per year, independently of HbA1c, age, and sex, whereas capillary density did not differ from that in controls over the same period (34). Müller cells and microglia in the retina also show reactive gliosis early in diabetes, exemplified by upregulated glial fibrillary acidic protein (GFAP) expression, and such changes can be detected in animal models within weeks after the induction of diabetes, earlier than clinically visible microaneurysms (5, 33). These findings indicate that retinal neurodegeneration is not merely a passive consequence of vascular injury, but rather a primary pathological process directly driven by metabolic abnormalities, and its timing provides an opportunity window for early neuroprotective intervention.

### 5.2 Multiple structural subclinical abnormalities can be detected in patients without clinically apparent retinopathy

Using high-resolution imaging techniques such as SS-OCT, da Silva et al. found that patients with T2D who had mild DR or no DR exhibited reduced macular retinal thickness and superficial capillary density relative to controls, and that these changes were associated with markers of diabetic kidney disease, suggesting that neurovascular injury and renal microvascular damage may progress in parallel during the NDR stage (3, 35). In a T2D cohort, Pedersen et al. reported that patients with MCI had thinner macular ganglion cell layers than those without MCI, whereas van Dijk et al. demonstrated that patients with T2D who had no DR or only minimal DR had thinning of the parafoveal RNFL, ganglion cell layer, and inner plexiform layer compared with controls, further supporting the neurodegenerative component of early DR (4, 36). More sensitive OCTA studies further indicate that enlargement of the foveal avascular zone (FAZ) and reduced density of the deep capillary plexus (DCP) are already present during the NDR stage, and that the spatial distribution of early capillary dropout overlaps closely with thinning of the inner retina (37, 38). In terms of functional assessment, multifocal electroretinography (mfERG) studies have shown prolonged local retinal response implicit times in patients with diabetes both with and without DR, and even those without retinopathy may exhibit subclinical localized dysfunction (39). Beyond mfERG, microperimetry, contrast sensitivity testing, and dark adaptation assessment have also detected abnormalities in patients with NDR. These functional tests can reveal neuronal impairment before structural changes become apparent, and their sensitivity may surpass that of OCT and fundus photography in certain contexts (39, 40). In addition, the earliest phase of DR may be dominated by different pathological pathways in different patients, including ischemia, neurodegeneration, or edema, of which ischemia appears most strongly correlated with disease severity (40). Together, these structural and functional phenotypes constitute the critical link between early metabolic abnormalities and clinically overt vascular disease (37, 41). The retina, as an extension of the central nervous system, shares a close pathophysiological connection between its microvascular changes and cerebral microvascular disease. Multiple epidemiological studies have shown that retinal neural layer thinning and reduced microvascular density are associated with imaging markers of cerebral small vessel disease, such as white matter hyperintensities and lacunar infarcts, suggesting that the retina may serve as a window for assessing cerebral microcirculation (4). Studies have found that patients with T2D and MCI have thinner retinal neural layers, and retinal microvascular abnormalities are also associated with cognitive decline and increased dementia risk. Glycemic variability may simultaneously damage retinal and cerebral microcirculation through repeated oxidative stress. As a marker of systemic microvascular injury, retinal microvascular abnormalities may contribute to the development of frailty syndromes in patients with diabetes, manifested as cognitive decline, reduced gait speed, and increased fall risk. Currently, research in this area consists largely of cross-sectional observations, and the causal relationship and temporal sequence between retinal microvascular markers and cerebral microvascular disease and cognitive outcomes require confirmation in prospective longitudinal studies.

### 5.3 The NDR stage represents the final critical window for retinal neuroprotection

Once retinal ganglion cells undergo apoptosis, the injury is irreversible. Mature RGCs lack intrinsic regenerative capacity within the central nervous system, and lost retinal neuronal function is difficult to restore therapeutically (5). Accordingly, identifying high-risk individuals during the NDR stage, when neurodegeneration has already been initiated but has not yet progressed to extensive irreversible cell loss, and introducing early intervention at this stage are crucial for reducing the blinding burden of DR. Achieving this goal requires clinical tools that can identify patients at high risk of metabolic stress during the NDR stage. CGM-derived GV metrics provide such a prospective risk signal: even when HbA1c is at target and no retinal vascular lesion is visible on fundus examination, persistently reduced TIR or persistently elevated CV may indicate that the individual is being exposed repeatedly to metabolic fluctuation-related injury to the NVU (21, 29). This temporal gap, in which metabolic warning precedes structural damage, underlies the concept that the NDR stage constitutes a key window for retinal neuroprotection. For early retinal neurodegeneration, currently available interventions can be considered on two levels. At the level of neuroprotection, the EUROCONDOR clinical trial evaluated topical somatostatin and brimonidine in patients with early or no DR; results showed that these two agents could arrest progression in patients with pre-existing neurodegeneration but had no preventive effect in those without neurodegeneration, highlighting the need to identify high-risk individuals before initiating neuroprotective therapy (51, 52). At the level of glycemic variability management, insulin pump systems integrated with CGM (closed-loop systems) can reduce intraday glucose fluctuations, and certain GLP-1 receptor agonists have demonstrated retinal neuroprotective and anti-inflammatory effects in animal models (53); however, the independent protective effects of these agents on early retinal injury in NDR populations have not yet been confirmed in prospective clinical trials.

## 6 Glycemic variability drives early retinal injury through oxidative stress and glial activation

### 6.1 Intermittent glycemic fluctuation induces stronger mitochondrial oxidative stress than sustained hyperglycemia

Intermittent glycemic fluctuation induces stronger mitochondrial oxidative stress than sustained hyperglycemia. Quagliaro et al. compared the effects of intermittent versus sustained high glucose in human umbilical vein endothelial cells and found that intermittent high glucose induced higher levels of nitrotyrosine, 8-hydroxydeoxyguanosine (a marker of oxidative DNA damage), and apoptosis than sustained high glucose, and that this process depended on protein kinase C (PKC)-mediated activation of NAD(P)H oxidase (42). Piconi et al. further showed that intermittent high glucose upregulated endothelial adhesion molecules, including intercellular adhesion molecule-1 (ICAM-1), vascular cell adhesion molecule-1 (VCAM-1), E-selectin, and interleukin-6 more strongly than sustained high glucose, and that this effect could be blocked by poly(ADP-ribose) polymerase (PARP) inhibition (11).

The molecular basis of these effects lies in mitochondrial electron transport chain disruption triggered by abrupt glucose transitions. When glucose concentrations rise abruptly from normal to high levels, electron flux through the mitochondrial electron transport chain increases sharply, resulting in the rapid burst release of superoxide anions. When glucose subsequently declines, downstream injury cascades that have already been triggered, including PKC activation and accelerated AGE formation, do not stop synchronously, thereby leaving a persistent “metabolic memory” effect within cells (18, 42). Monnier et al. validated this mechanism *in vivo* by showing that, in patients with T2D, 24-hour urinary free 8-iso-prostaglandin F2α was much more strongly correlated with glucose variability, measured by MAGE (r=0.86), than with HbA1c or mean glucose, suggesting that MAGE is an important predictor of systemic oxidative stress (10). In neurons such as RGCs, which are highly sensitive to oxidative stress, repeated acute glucose surges may lead to excessive accumulation of mitochondrial reactive oxygen species (ROS), trigger caspase-dependent apoptotic pathways, and ultimately cause neuronal loss (5). The above evidence indicates that the amplitude of glycemic variability captured by CGM (e.g., elevated CV and MAGE) may directly reflect the intensity of mitochondrial oxidative stress, providing a quantifiable risk indicator of the degree of metabolic stress imposed on retinal neurons during the NDR stage.

### 6.2 Glycemic variability activates Müller cells and microglia, creating a pro-inflammatory retinal microenvironment

Retinal Müller cells undergo reactive activation under glycemic fluctuation. Müller cells are the most abundant macroglial cells in the retina and play supportive roles in maintaining NVU metabolic homeostasis, regulating extracellular glutamate levels, and preserving BRB integrity (5, 43). Under diabetic or hyperglycemic conditions, Müller cells undergo reactive activation characterized by upregulation of GFAP and vimentin, cellular swelling, and reduced glutamine synthetase activity, which in turn diminishes extracellular glutamate clearance and enhances excitotoxicity (43). This reactive state is accompanied by increased transcription and secretion of pro-inflammatory mediators, including tumor necrosis factor-α (TNF-α), interleukin-1β (IL-1β), and interleukin-6 (IL-6), while neurotrophic support is correspondingly weakened. Because glycemic fluctuations induce stronger oxidative stress than sustained hyperglycemia, repeated fluctuations may further amplify this glial response (43).

Microglia activate in parallel during early diabetes, together with Müller cells forming a positive-feedback neuroinflammatory cascade. Retinal microglia, the resident innate immune cells of the retina, shift early in diabetes from a ramified surveillance phenotype to an amoeboid reactive phenotype, releasing ROS, nitric oxide, and pro-inflammatory mediators through pathways such as nuclear factor-κB (NF-κB), and this shift has been linked to RNFL thinning (44). Activated Müller cells and microglia together create a local inflammatory microenvironment: pro-inflammatory mediators exacerbate RGC apoptosis through paracrine effects, while dying neurons release damage-associated molecular patterns that activate additional glial cells, forming a positive-feedback neuroinflammatory cascade (44). This glial activation-centered process constitutes a major cellular pathological basis through which glycemic variability drives retinal neurodegeneration. This glial response is already initiated during the NDR stage, and its amplification is closely related to the frequency and amplitude of glycemic fluctuations, explaining why patients with persistently low TIR on CGM are more likely to exhibit subclinical retinal neurodegeneration.

### 6.3 Glycemic variability disrupts endothelial tight junctions and triggers early blood-retinal barrier leakage

Recurrent glycemic fluctuation directly damages BRB tight junction structures. The BRB consists of tight junctions between retinal capillary endothelial cells together with the retinal pigment epithelium and is essential for maintaining the microenvironmental homeostasis of retinal neural tissue (2, 45). Repeated hyperglycemic surges activate PKC (see Section 6.1) and the NADPH oxidase pathway, leading to sustained superoxide production and causing disordered membrane localization, reduced expression, and accelerated internalization of tight junction and adhesion proteins such as claudin-5, occludin, and vascular endothelial cadherin (VE-cadherin) (45).

**Figure 3:**
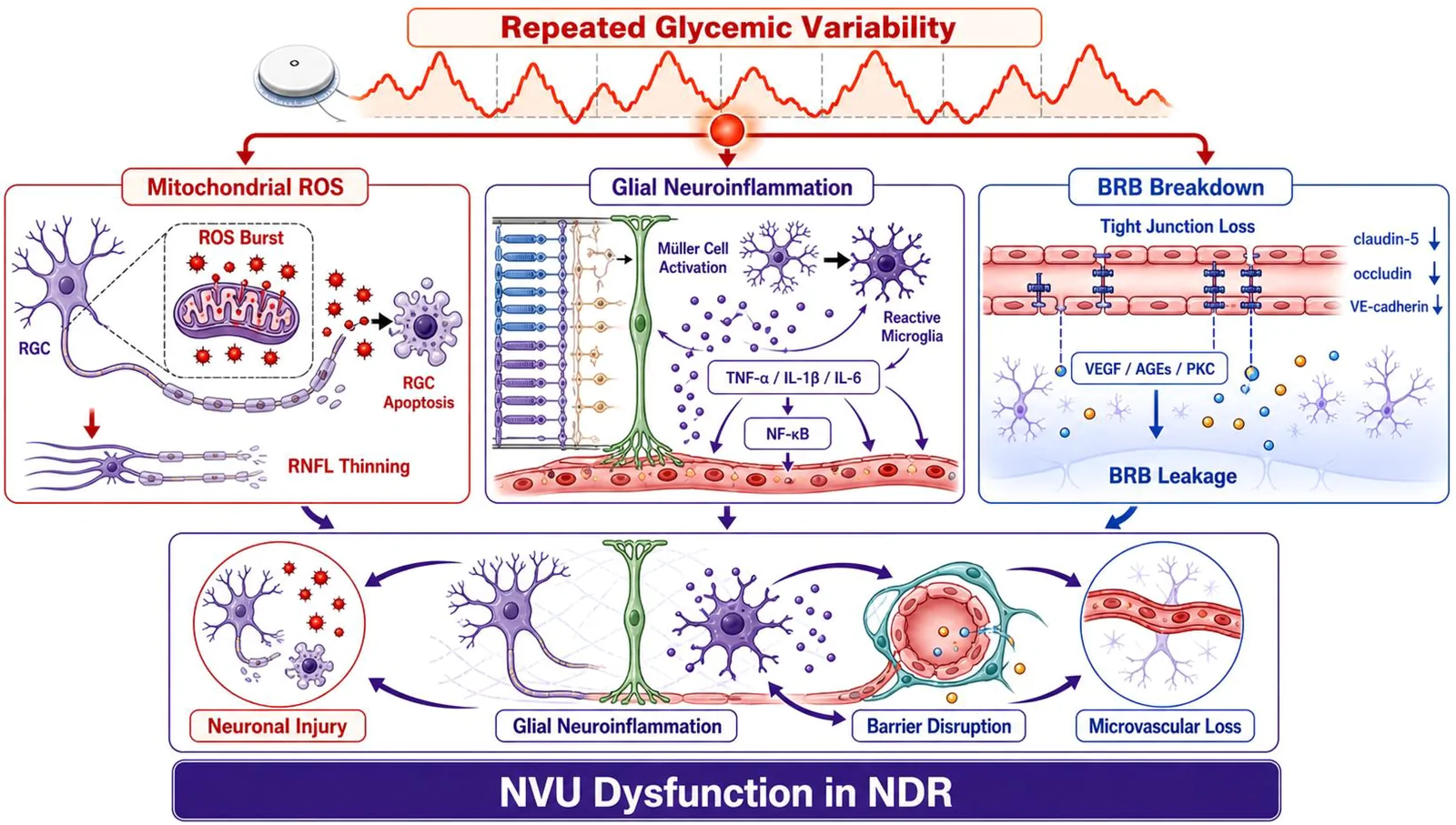
Molecular mechanisms by which glycemic variability drives retinal neurovascular unit injury. Recurrent glycemic fluctuations may promote retinal injury during the NDR stage through multiple mutually amplifying pathways, including excessive mitochondrial ROS generation and RGC apoptosis, activation of Müller cells and microglia to establish a pro-inflammatory retinal microenvironment, and disruption of BRB tight-junction proteins with consequent early leakage. Together, these processes lead to NVU dysfunction, neural layer thinning, and reduced microvascular density.

BRB injury, glial activation, and AGE accumulation form a mutually amplifying injury loop. Activated Müller cells also secrete increased vascular endothelial growth factor (VEGF), which further disrupts endothelial cell-cell junctions; even subclinical elevations in VEGF may be sufficient to significantly increase vascular permeability (43, 45). Under conditions of glycemic variability, accelerated generation of AGE precursors such as methylglyoxal during glucose peaks (as described in Section 6.1) modifies tight junction proteins and extracellular matrix components, thereby damaging BRB structure and function at multiple levels (45). BRB leakage permits plasma proteins and inflammatory mediators to enter retinal neural tissue, leading to local edema, ischemia, and secondary neuronal injury, and establishing an amplifying injury loop involving vascular leakage, inflammatory infiltration, and secondary neural apoptosis (2, 45). Recurrent early injury to the BRB is therefore a key link connecting metabolic abnormalities to retinal structural damage. An integrated schematic of these mechanisms is presented in **Figure 3**. Recurrent BRB injury from glycemic fluctuation occurs before clinically visible vascular exudation, suggesting that patients with NDR who show elevated CV on CGM may already be in a subclinical phase of barrier dysfunction, supporting the inclusion of CGM-derived GV metrics in early risk assessment.

## 7 Performance of CGM-derived glycemic variability metrics in association with early retinal injury

### 7.1 Cross-sectional and prospective cohort studies support the independent informational value of CGM-derived glycemic variability beyond HbA1c

In studies of patients with T2D and LADA, Lu et al. used 72-hour CGM to assess glucose variability and found that variability measured by SD was independently associated with DR in T2D, even after adjustment for HbA1c, diabetes duration, and other conventional risk factors (20). Picconi et al. further reported in T1D that glucose variability was an independent predictor of early retinal neural structural changes, separate from the influence of HbA1c, thereby suggesting that glucose variability provides incremental information beyond HbA1c (21). In the T1D setting, De Meulemeester et al. studied 808 patients and compared TIR (3.9-10.0 mmol/L) and TITR (3.9-7.8 mmol/L) with microvascular complications; they found that higher TITR was associated with a lower prevalence of DR and that this association remained significant after adjustment for HbA1c (29). Cross-sectional studies by Lu et al. and Wang et al. likewise suggested that each 10% decrease in TIR was associated with an increased risk of DR in T2D, with Wang et al. reporting an increase of approximately 8% (46, 47). More prospective evidence was provided by Wang et al., who followed 2,518 adults with T2D and no baseline DR for approximately 5.4 years and found that each 10% decrease in TITR was associated with a 9% increase in incident DR risk (HR 1.09, 95% CI 1.06-1.13) (48). Analysis of the DCCT virtual CGM dataset by Lobo et al. further showed that the strengths of association between TIR, TITR, and retinopathy progression endpoints were comparable to that of HbA1c, indicating that the three may complement one another (30). The international consensus led by Beck et al. has already established TIR as a valid outcome measure for diabetes clinical trials (24). In addition, the analysis by Vigersky and McMahon showed a strong linear inverse relationship between TIR and HbA1c (R≈-0.84), with each 10% difference in TIR corresponding to approximately a 0.8% difference in HbA1c; however, the wide range of TIR values observed at a given HbA1c level indicates that the two metrics provide complementary rather than redundant information (6). Anagnostopoulou et al. concluded in their review that CGM-derived TIR, GV, and TITR provide supplementary information beyond HbA1c in predicting DR and other microvascular complications and may be more sensitive to early subclinical injury (31). From a cross–diabetes-type perspective, the association between GV and early retinal injury has been observed in both T1D and T2D (20, 21, 29, 48), suggesting a degree of universality; however, current prospective evidence (e.g., the incident DR cohort by Wang et al. (48)) comes only from T2D populations, whereas T1D evidence remains predominantly cross-sectional. The association between glycemic variability and retinal injury in LADA has not been independently validated, as existing data derive from mixed cohorts that include LADA (20). With regard to susceptible subgroups, patients with T2D and concomitant diabetic kidney disease show more pronounced reductions in macular retinal thickness and capillary density (3), while those with mild cognitive impairment have thinner retinal ganglion cell layers (4), suggesting that subgroups with combined microvascular injury and a propensity for systemic neurodegeneration may be more sensitive to GV. Patients with long-standing T1D, whose retinal neurons have diminished compensatory capacity due to prolonged exposure to metabolic stress, may also represent a high-risk subgroup, but CGM–retinal longitudinal data specific to this population are lacking. The precise relationship between GV and retinal injury in these subgroups requires future stratified studies targeting specific diabetes types and comorbidities.

### 7.2 The potential of TIR and CV in assessing the risk of early retinal neural injury

Compared with single time-based metrics such as TAR or TBR, TIR reflects the total time spent within the metabolically safe range and implicitly captures information related to both hyperglycemia and hypoglycemia (14). In analyses based on the DCCT virtual CGM dataset, TIR and TITR showed associations with retinopathy progression that were comparable in strength to HbA1c, and TITR performed similarly to TIR, with both metrics being straightforward to interpret and translate into clinical targets (30). From the perspective of amplitude-based metrics, CV, for which international consensus recommends a threshold of ≤36%, has already shown independent value in predicting cutaneous microvascular endothelial dysfunction, consistent with the concept that glycemic fluctuations induce acute oxidative stress (10). Existing evidence suggests that TIR may serve as a time-based biomarker of DR risk, whereas CV may serve as an amplitude-based indicator of fluctuation severity. Their combined use may therefore enable risk stratification for early retinal injury in patients with NDR (29, 31). Nevertheless, the optimal risk assessment thresholds of TIR and CV for early retinal abnormalities in different diabetes populations, including early T1D, early T2D, different age groups, and different ethnic groups, remain to be calibrated. Although existing evidence supports the value of CGM-derived GV metrics in the early DR risk assessment, the field remains at an early stage and has several limitations. From the standpoint of sample size and study design, most key studies have been cross-sectional, and sufficiently robust longitudinal prospective data confirming the causal sequence and temporal priority between abnormal glycemic variability and retinal neurodegeneration remain lacking (31). At the level of CGM standardization, different studies have used different devices, including Medtronic Guardian, Dexcom G6/G7, and Abbott FreeStyle Libre systems, and monitoring durations have ranged from 72 hours to 14 days, without a unified protocol (7). At the level of OCT standardization, systematic differences exist among devices such as Zeiss Cirrus, Topcon Maestro, and Heidelberg Spectralis in their measurement algorithms and reference ranges for macular ganglion cell layer thickness, RNFL thickness, and vascular density, which hinders the direct integration and comparison of multicenter data (3). With respect to confounding control, existing studies have not applied uniform adjustment strategies for key factors such as diabetes duration, baseline HbA1c, blood pressure, and lipid control, which affects the precise estimation of the independent effect size linking glycemic variability to retinal injury (31). These limitations mean that current evidence remains more suggestive than definitive and should be addressed systematically in future large-scale, multicenter, prospective cohort studies.

## 8 Incorporating CGM-derived glycemic variability into screening systems may enable precise and individualized management of diabetic eye disease

### 8.1 Incorporating CGM-derived glycemic variability into early screening strategies for diabetic eye disease may shift the intervention point forward

Based on current evidence, patients with NDR who show marked glycemic variability on CGM, particularly those with TIR <70% or CV >36%, should be regarded as a high-risk subgroup for retinal neural injury even if their HbA1c remains within target range. Such individuals may warrant intensified monitoring, including periodic assessment of neural layer thickness and microvascular density using OCT and OCTA (3, 14). The core idea is to shift DR screening from a passive model that depends on fundus photography to detect vascular lesions toward an active identification model in which CGM-derived risk signals guide OCT-based neural layer assessment, thereby identifying metabolically high-stress subgroups during the NDR stage and introducing early neuroprotective intervention (5). From a health-economic perspective, as CGM devices become less expensive and more widely accessible, the incremental cost of incorporating them into early screening is likely to become increasingly acceptable. In subgroups at high risk of DR-related blindness, such as those with long-standing T1D or marked glycemic variability in T2D, this forward-looking screening strategy may reduce blindness rates and lower the long-term healthcare burden related to diabetes (1, 7). Combining CGM-derived GV metrics with functional retinal assessments such as mfERG may enable simultaneous capture of metabolic stress severity and early neuronal functional impairment during the NDR stage, offering a more effective approach to identifying early DR neural damage than either modality alone and providing a more refined assessment dimension for stratified screening.

### 8.2 Building a dual-dimensional “HbA1c + TIR/CV” risk assessment strategy may support precise individualized management

Current diabetes management guidelines still place HbA1c at the center of glycemic assessment, yet ample evidence indicates that patients with the same HbA1c may exhibit different patterns of glycemic variability and therefore different complication risks (6, 20). A future dual-dimensional risk assessment framework combining HbA1c with TIR/CV may therefore be useful. In this framework, HbA1c provides a macroscopic assessment of long-term average glycemic control, whereas TIR and CV provide dynamic information regarding metabolic stability and the degree of glycemic fluctuation; together, they offer a more complete picture of glycemic control quality in each patient.

**Figure 4:**
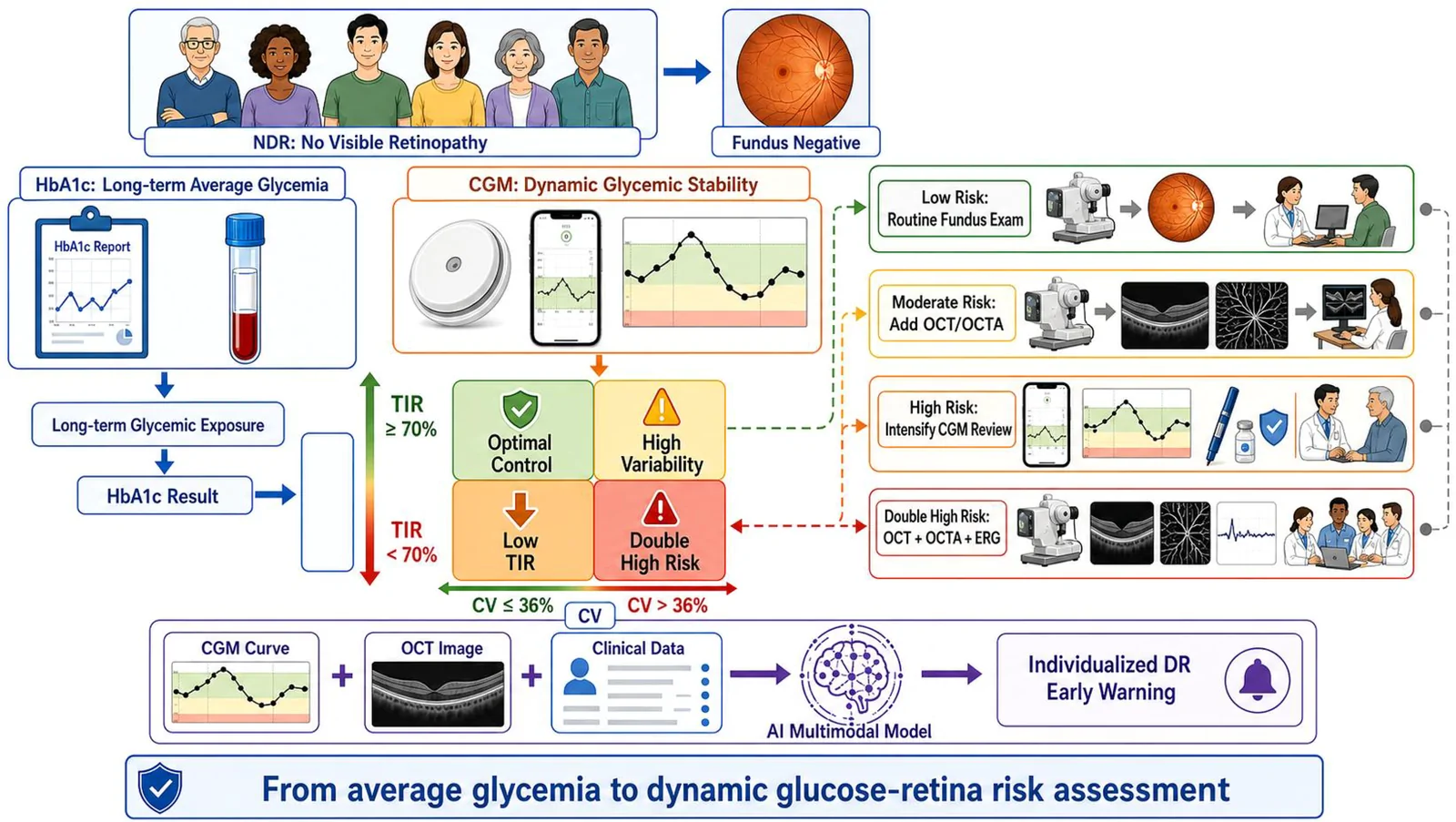
Dual-dimensional risk stratification and screening pathway based on “HbA1c + TIR/CV.” HbA1c reflects long-term average glycemic control, whereas TIR and CV provide information on exposure time within target range and the amplitude of glycemic fluctuation, respectively. Integration of these parameters may enable the classification of patients with NDR into different risk strata, thereby guiding the frequency of OCT/OCTA/electroretinography assessment, follow-up intervals, and the intensity of glycemic management. This strategy may help shift the intervention point for DR from the stage of clinically visible vascular lesions to the stage of subclinical neurovascular unit injury.

Operationally, patients could be classified into four groups according to TIR and CV values, for example: TIR ≥70% and CV ≤36% as good control; TIR ≥70% and CV >36% as acceptable average control but marked variability; TIR <70% and CV ≤36% as poor average control but relatively stable fluctuation; and TIR <70% and CV >36% as dual high risk. Evidence-based differences in retinal screening frequency and indications for neural layer assessment could then be assigned to each subgroup (29, 31). Such a stratification strategy may optimize the allocation of medical resources by reducing unnecessary OCT examinations in low-risk individuals while ensuring timely identification and early intervention in high-risk patients. A schematic pathway for dual-dimensional risk stratification and screening based on “HbA1c + TIR/CV” is shown in **Figure 4**.

In practical application, TIR <70% or CV >36% within the above four-group classification corresponds to the high-risk subgroup; however, it should be noted that these thresholds are derived from international consensus recommendations for T1D and T2D (14) and have not been validated in prospective studies of NDR-specific populations. Patients with T1D typically have greater glycemic fluctuation amplitudes and higher baseline CV than those with T2D, while LADA falls between the two, suggesting that high-risk thresholds may need to be differentially set according to diabetes type. With regard to CGM monitoring frequency, given cost and accessibility considerations, performing CGM assessment for 7–14 days every 3–6 months is recommended for patients with NDR to balance cost accessibility and data representativeness. It should be emphasized that the current dual-dimensional strategy is primarily a conceptual framework based on existing evidence, and its clinical effectiveness requires validation in large-scale prospective cohorts and interventional trials before it can be incorporated into formal guidelines.

When CGM identifies marked glycemic variability, clinical management can be adjusted accordingly. For patients with T1D or long-standing T2D using insulin, insulin pump systems integrated with CGM (closed-loop systems) can automatically adjust basal and prandial insulin delivery based on real-time glucose trends, effectively reducing intraday glycemic fluctuations. Among newer antidiabetic agents, GLP-1 receptor agonists reduce postprandial glucose excursions by delaying gastric emptying and stimulating glucose-dependent insulin secretion, while SGLT-2 inhibitors lower glycemic load by promoting urinary glucose excretion, and both classes are beneficial for improving TIR and CV (54). Dual receptor agonists targeting both GLP-1 and GIP (e.g., tirzepatide) may confer additional benefits in reducing glycemic variability, although their impact on retinal microcirculation remains to be evaluated. Improving insulin resistance (e.g., with metformin or thiazolidinediones) can lower overall glucose levels and indirectly reduce the amplitude of glycemic fluctuation. Lifestyle interventions (regular exercise, adjusting dietary structure and meal sequencing) have an immediate effect on improving postprandial glucose peaks (54). The efficacy of these therapeutic approaches in reducing GV is supported by CGM-metric-quantified evidence, but whether these reductions translate into protective effects against early retinal neural injury in NDR populations requires prospective studies for confirmation.

The management targets for glycemic variability need to be set separately according to the specific comorbidities of each patient. Reducing GV can confer retinal neuroprotective benefits, but patients with different comorbidities face different safety constraints when achieving these benefits. Patients with concomitant CKD are at significantly elevated risk of hypoglycemic events due to reduced renal gluconeogenesis and delayed clearance of hypoglycemic agents, and severe hypoglycemia itself is associated with worsening of retinal neural injury; therefore, in patients with CKD, prioritizing TBR <4% (severe hypoglycemia <1%) is more critical than achieving CV targets, and GV management should follow a gradual strategy of “first reduce hypoglycemia, then optimize fluctuation” (14, 55). In patients with concomitant ASCVD, acute postprandial hyperglycemic spikes directly increase cardiovascular event risk by inducing endothelial dysfunction and a prothrombotic state, while glucose peaks are also the primary triggers of retinal oxidative stress; reducing TAR and CV in these patients is protective for both cardiovascular and retinal outcomes, favoring more aggressive glycemic fluctuation control (56). For elderly patients or those with autonomic neuropathy, impaired hypoglycemia awareness makes aggressive glucose lowering particularly hazardous; in these populations, CGM serves not only as a GV assessment tool but also as a safety net for detecting asymptomatic hypoglycemia, and the intensity of GV management should be advanced gradually under real-time CGM feedback (57). Therefore, in the absence of large RCTs specifically targeting GV management in NDR patients, the intensity of GV control should be matched to each patient’s hypoglycemia tolerance and specific comorbidities, rather than applying a uniform threshold.

### 8.3 Large prospective longitudinal cohorts and randomized interventional trials are necessary for future development

To incorporate CGM-derived GV metrics into formal early DR screening guidelines, research must advance in the following directions. First, large-scale, multicenter, prospective longitudinal cohorts are needed to establish the independent associative capacity and optimal risk assessment thresholds of TIR and CV for early retinal neural layer thinning and long-term DR progression across different diabetes types (T1D, T2D, and LADA), age groups, and ethnic populations (31). On this basis, randomized controlled trials (RCTs) are needed to determine whether glycemic management strategies specifically targeting improved TIR and reduced CV can slow early retinal neural layer thinning or delay the transition from NDR to clinically apparent DR (30).

In addition, OCT and OCTA devices require the establishment of unified scanning protocols, segmentation algorithms, and normal reference ranges, or the use of artificial intelligence-assisted automated annotation to reduce inter-device systematic error, thereby enabling the integration of multicenter CGM-retinal structural longitudinal data. For screening pathway design, CGM could serve as a first-tier stratification tool, triaging patients with NDR according to the degree of GV and performing OCT/OCTA and mfERG depth assessments only for high-risk individuals (TIR <70% or CV >36%); this “CGM first, imaging follows” stepped pathway may ensure that high-risk individuals are not missed while controlling cost (2, 15). Progress in these directions is essential for transforming CGM-derived GV from a suggestive risk signal into a standardized clinical screening indicator (31).

## 9 Limitations

The current evidence and this review have the following limitations: first, most key studies are cross-sectional or observational in design with relatively small sample sizes, and the longitudinal mechanistic-clinical linkage is limited; the causal relationship between abnormal glycemic variability and retinal neurodegeneration requires confirmation in prospective studies. Second, CGM technologies, data quality, and definitions of GV metrics vary across studies, and OCT/OCTA measurement algorithms are not yet unified, affecting result comparability. Third, data from truly NDR populations and from Asian, African, and other non-Western cohorts are limited, and the generalizability of current conclusions requires validation. Fourth, there are no interventional trials specifically targeting improved GV to prevent retinal neurodegeneration. Furthermore, as this is a narrative review, no formal quality assessment was performed, and selection bias may exist. Nevertheless, existing evidence consistently suggests an independent association between glycemic variability and early retinal injury, and these limitations provide clear directions for improvement in the design of future large-scale, multicenter, prospective studies.

## 10 Conclusions

This review summarizes the clinical evidence and molecular mechanisms linking CGM-derived glycemic variability metrics to their association with early retinal neurovascular injury in patients with NDR. As discussed above, HbA1c cannot adequately reflect the dynamic features of day-to-day glucose fluctuation and may obscure the risk of early retinal injury driven by metabolic instability in patients whose HbA1c is considered well controlled. CGM-derived metrics, particularly TIR and CV, capture both the time-based and amplitude-based dimensions of glycemic dynamics and can provide statistically meaningful incremental information on retinal injury risk beyond the HbA1c average (49). Mechanistically, glycemic variability damages the retinal NVU at molecular, cellular, and structural levels through acute mitochondrial oxidative stress that is more severe than that induced by sustained hyperglycemia, Müller cell- and microglia-mediated neuroinflammation, and repeated disruption of BRB tight-junction proteins (50). These injuries begin to accumulate during the NDR stage, before overt vascular lesions are visible on fundus examination, and can be detected *in vivo* by OCT, OCTA, and electroretinography. Therefore, integrating CGM-derived metrics such as TIR and CV with high-resolution retinal imaging may enable the establishment of an early warning and risk stratification system for early retinal neurovascular injury during the NDR stage, providing a new clinical window for precise intervention and reshaping the strategic framework for the early prevention and treatment of diabetic eye disease.

## References

[1] Fung TH Patel B Wilmot EG Amoaku WM . Diabetic retinopathy for the non-ophthalmologist. Clin Med (Lond). (2022) 22:112–6. doi: 10.7861/clinmed.2021-0792 35304370 PMC8966825

[2] Seo H Park SJ Song M . Diabetic retinopathy (DR): mechanisms, current therapies, and emerging strategies. Cells. (2025) 14:376. doi: 10.3390/cells14050376 40072104 PMC11898816

[3] da Silva MO do Carmo Chaves AEC Gobbato GC dos Reis MA Lavinsky F Schaan BD . Early neurovascular retinal changes detected by swept-source OCT in type 2 diabetes and association with diabetic kidney disease. Int J Retina Vitreous. (2021) 7:73. doi: 10.1186/s40942-021-00347-z 34865654 PMC8647413

[4] Pedersen FN Stokholm L Lois N Yang D Cheung CY Biessels GJ . Structural and metabolic retinal changes associated with mild cognitive impairment in type 2 diabetes. Diabetes. (2023) 72:1853–63. doi: 10.2337/db23-0025 37725903

[5] Antonetti DA Barber AJ Bronson SK Freeman WM Gardner TW Jefferson LS . Diabetic retinopathy: seeing beyond glucose-induced microvascular disease. Diabetes. (2006) 55:2401–11. doi: 10.2337/db05-1635 16936187

[6] Vigersky RA McMahon C . The relationship of hemoglobin A1C to time-in-range in patients with diabetes. Diabetes Technol Ther. (2019) 21:81–5. doi: 10.1089/dia.2018.0310 30575414

[7] Yapanis M James S Craig ME O'Neal D Ekinci EI . Complications of diabetes and metrics of glycemic management derived from continuous glucose monitoring. J Clin Endocrinol Metab. (2022) 107:e2221–36. doi: 10.1210/clinem/dgac034 35094087 PMC9113815

[8] Psoma O Makris M Tselepis A Tsimihodimos V . Short-term glycemic variability and its association with macrovascular and microvascular complications in patients with diabetes. J Diabetes Sci Technol. (2024) 18:956–67. doi: 10.1177/19322968221146808 36576014 PMC11307209

[9] Hirsch IB . Glycemic variability and diabetes complications: does it matter? Of course it does! Diabetes Care. (2015) 38:1610–4. doi: 10.2337/dc14-2898 26207054

[10] Monnier L Mas E Ginet C Michel F Villon L Cristol JP . Activation of oxidative stress by acute glucose fluctuations compared with sustained chronic hyperglycemia in patients with type 2 diabetes. JAMA. (2006) 295:1681–7. doi: 10.1001/jama.295.14.1681 16609090

[11] Piconi L Quagliaro L Da Ros R Assaloni R Giugliano D Esposito K . Intermittent high glucose enhances ICAM-1, VCAM-1, E-selectin and interleukin-6 expression in human umbilical endothelial cells in culture: the role of poly(ADP-ribose) polymerase. J Thromb Haemost. (2004) 2:1453–9. doi: 10.1111/j.1538-7836.2004.00835.x 15304054

[12] Huang L Pan Y Zhou K Liu H Zhong S . Correlation between glycemic variability and diabetic complications: a narrative review. Int J Gen Med. (2023) 16:3083–94. doi: 10.2147/ijgm.s418520 37496596 PMC10368016

[13] Brownlee M Hirsch IB . Glycemic variability: a hemoglobin A1c-independent risk factor for diabetic complications. JAMA. (2006) 295:1707–8. doi: 10.1001/jama.295.14.1707 16609094

[14] Battelino T Danne T Bergenstal RM Amiel SA Beck R Biester T . Clinical targets for continuous glucose monitoring data interpretation: recommendations from the International Consensus on Time in Range. Diabetes Care. (2019) 42:1593–603. doi: 10.2337/dci19-0028 31177185 PMC6973648

[15] Tecce N Cennamo G Rinaldi M Costagliola C Colao A . Exploring the impact of glycemic control on diabetic retinopathy: emerging models and prognostic implications. J Clin Med. (2024) 13:831. doi: 10.3390/jcm13030831 38337523 PMC10856421

[16] Danne T Nimri R Battelino T Bergenstal RM Close KL DeVries JH . International consensus on use of continuous glucose monitoring. Diabetes Care. (2017) 40:1631–40. doi: 10.2337/dc17-1600 29162583 PMC6467165

[17] McCarter RJ Hempe JM Gomez R Chalew SA . Biological variation in HbA1c predicts risk of retinopathy and nephropathy in type 1 diabetes. Diabetes Care. (2004) 27:1259–64. doi: 10.2337/diacare.27.6.1259 15161772

[18] Ceriello A Esposito K Piconi L Ihnat MA Thorpe JE Testa R . Oscillating glucose is more deleterious to endothelial function and oxidative stress than mean glucose in normal and type 2 diabetic patients. Diabetes. (2008) 57:1349–54. doi: 10.2337/db08-0063 18299315

[19] Nathan DM Genuth S Lachin J Cleary P Crofford O Davis M . The effect of intensive treatment of diabetes on the development and progression of long-term complications in insulin-dependent diabetes mellitus. N Engl J Med. (1993) 329:977–86. doi: 10.1056/NEJM199309303291401 8366922

[20] Lu J Ma X Zhang L Mo Y Ying L Lu W . Glycemic variability assessed by continuous glucose monitoring and the risk of diabetic retinopathy in latent autoimmune diabetes of the adult and type 2 diabetes. J Diabetes Investig. (2019) 10:753–9. doi: 10.1111/jdi.12957 30306722 PMC6497773

[21] Picconi F Parravano M Ylli D Pasqualetti P Coluzzi S Giordani I . Retinal neurodegeneration in patients with type 1 diabetes mellitus: the role of glycemic variability. Acta Diabetol. (2017) 54:489–97. doi: 10.1007/s00592-017-0971-4 28238189 PMC5385321

[22] Shah VN DuBose SN Li Z Beck RW Peters AL Weinstock RS . Continuous glucose monitoring profiles in healthy nondiabetic participants: a multicenter prospective study. J Clin Endocrinol Metab. (2019) 104:4356–64. doi: 10.1210/jc.2018-02763 31127824 PMC7296129

[23] Suh S Kim JH . Glycemic variability: how do we measure it and why is it important? Diabetes Metab J. (2015) 39:273–82. doi: 10.4093/dmj.2015.39.4.273 26301188 PMC4543190

[24] Beck RW Bergenstal RM Riddlesworth TD Kollman C Li Z Brown AS . Validation of time in range as an outcome measure for diabetes clinical trials. Diabetes Care. (2019) 42:400–5. doi: 10.2337/dc18-1444 30352896 PMC6905478

[25] Ceriello A Kilpatrick ES . Glycemic variability: both sides of the story. Diabetes Care. (2013) 36:S272–5. doi: 10.2337/dcs13-2030 23882058 PMC3920802

[26] Wright LA Hirsch IB . Metrics beyond hemoglobin A1C in diabetes management: time in range, hypoglycemia, and other parameters. Diabetes Technol Ther. (2017) 19:S16–26. doi: 10.1089/dia.2017.0029 28541136 PMC5444503

[27] Kovatchev BP . Metrics for glycaemic control - from HbA1c to continuous glucose monitoring. Nat Rev Endocrinol. (2017) 13:425–36. doi: 10.1038/nrendo.2017.3 28304392

[28] Rodbard D . Clinical interpretation of indices of quality of glycemic control and glycemic variability. Postgrad Med. (2011) 123:107–18. doi: 10.3810/pgm.2011.07.2310 21680995

[29] De Meulemeester J Charleer S Visser MM De Block C Mathieu C Gillard P . The association of chronic complications with time in tight range and time in range in people with type 1 diabetes: a retrospective cross-sectional real-world study. Diabetologia. (2024) 67:1527–35. doi: 10.1007/s00125-024-06171-y 38787436

[30] Lobo B Kanapka L Kovatchev BP Kollman C Beck RW . The association of time-in-range and time-in-tight-range with retinopathy progression in the virtual diabetes control and complications trial continuous glucose monitoring dataset. Diabetes Technol Ther. (2025) 27:558–61. doi: 10.1089/dia.2025.0033 39989301

[31] Anagnostopoulou L Liarakos AL Ntanasis-Stathopoulos I Briasoulis A Tentolouris A . Continuous glucose monitoring and microvascular complications in diabetes: bridging glycemic metrics with clinical outcomes. Diabetes Obes Metab. (2026) 28:840–9. doi: 10.1111/dom.70288 41208627 PMC12803653

[32] Jonsson KB Frydkjaer-Olsen U Grauslund J . Vascular changes and neurodegeneration in the early stages of diabetic retinopathy: which comes first? Ophthalmic Res. (2016) 56:1–9. doi: 10.1159/000444498 27035578

[33] Simó R Stitt AW Gardner TW . Neurodegeneration in diabetic retinopathy: does it really matter? Diabetologia. (2018) 61:1902–12. doi: 10.1007/s00125-018-4692-1 PMC6096638 30030554

[34] Sohn EH van Dijk HW Jiao C Kok PHB Jeong W Demirkaya N . Retinal neurodegeneration may precede microvascular changes characteristic of diabetic retinopathy in diabetes mellitus. Proc Natl Acad Sci USA. (2016) 113:E2655–64. doi: 10.1073/pnas.1522014113 27114552 PMC4868487

[35] Vujosevic S Midena E . Retinal layers changes in human preclinical and early clinical diabetic retinopathy support early retinal neuronal and Müller cells alterations. J Diabetes Res. (2013) 2013:905058. doi: 10.1155/2013/905058 23841106 PMC3694491

[36] van Dijk HW Verbraak FD Kok PHB Stehouwer M Garvin MK Sonka M . Early neurodegeneration in the retina of type 2 diabetic patients. Invest Ophthalmol Vis Sci. (2012) 53:2715–9. doi: 10.1167/iovs.11-8997 22427582 PMC3366721

[37] Kim K Kim ES Yu SY . Optical coherence tomography angiography analysis of foveal microvascular changes and inner retinal layer thinning in patients with diabetes. Br J Ophthalmol. (2018) 102:1226–31. doi: 10.1136/bjophthalmol-2017-311149 29259019

[38] Cutruzzola A Carnevali A Gatti V Latella G Lamonica L Oliverio F . Continuous glucose monitoring-derived metrics and capillary vessel density in subjects with type 1 diabetes without diabetic retinopathy. J Diabetes Res. (2023) 2023:9516059. doi: 10.1155/2023/9516059 37096234 PMC10122598

[39] Fortune B Schneck ME Adams AJ . Multifocal electroretinogram delays reveal local retinal dysfunction in early diabetic retinopathy. Invest Ophthalmol Vis Sci. (1999) 40:2638–51. 10509661

[40] Marques IP Alves D Santos T Mendes L Santos AR Lobo C . Multimodal imaging of the initial stages of diabetic retinopathy: different disease pathways in different patients. Diabetes. (2019) 68:648–53. doi: 10.2337/db18-1077 30523027

[41] Lasta M Pemp B Schmidl D Boltz A Kaya S Palkovits S . Neurovascular dysfunction precedes neural dysfunction in the retina of patients with type 1 diabetes. Invest Ophthalmol Vis Sci. (2013) 54:842–7. doi: 10.1167/iovs.12-10873 23307962

[42] Quagliaro L Piconi L Assaloni R Martinelli L Motz E Ceriello A . Intermittent high glucose enhances apoptosis related to oxidative stress in human umbilical vein endothelial cells: the role of protein kinase C and NAD(P)H-oxidase activation. Diabetes. (2003) 52:2795–804. doi: 10.2337/diabetes.52.11.2795 14578299

[43] Coughlin BA Feenstra DJ Mohr S . Müller cells and diabetic retinopathy. Vision Res. (2017) 139:93–100. doi: 10.1016/j.visres.2017.03.013 28866025 PMC5794018

[44] Altmann C Schmidt MHH . The role of microglia in diabetic retinopathy: inflammation, microvasculature defects and neurodegeneration. Int J Mol Sci. (2018) 19:110. doi: 10.3390/ijms19010110 29301251 PMC5796059

[45] Klaassen I Van Noorden CJ Schlingemann RO . Molecular basis of the inner blood-retinal barrier and its breakdown in diabetic macular edema and other pathological conditions. Prog Retin Eye Res. (2013) 34:19–48. doi: 10.1016/j.preteyeres.2013.02.001 23416119

[46] Lu J Ma X Zhou J Zhang L Mo Y Ying L . Association of time in range, as assessed by continuous glucose monitoring, with diabetic retinopathy in type 2 diabetes. Diabetes Care. (2018) 41:2370–6. doi: 10.2337/dc18-1131 30201847

[47] Wang Y Lu J Shen Y Ni J Zhang L Lu W . Comparison of glucose time in range and area under curve in range in relation to risk of diabetic retinopathy in type 2 diabetes patients. J Diabetes Investig. (2022) 13:1543–50. doi: 10.1111/jdi.13811 35435323 PMC9434583

[48] Wang Y Lu J Yu J Ni J Wang M Lu W . Association between time in tight range and incident diabetic retinopathy in adults with type 2 diabetes. Diabetes Obes Metab. (2025) 27:1415–22. doi: 10.1111/dom.16143 39696843 PMC11802404

[49] Chen B Shen C Sun B . Current landscape and comprehensive management of glycemic variability in diabetic retinopathy. J Transl Med. (2024) 22:700. doi: 10.1186/s12967-024-05516-w 39075573 PMC11287919

[50] Maran JJ Kuo CYJ Rupenthal ID Murphy R Mugisho OO . Serum albumin and glycemic variability could contribute to diabetic retinopathy progression by regulating chronic inflammatory pathways. J Ophthalmol. (2025) 2025:9673736. doi: 10.1155/joph/9673736 41497728 PMC12767037

[51] Simo R Hernandez C Porta M Bandello F Grauslund J Harding SP . Effects of topically administered neuroprotective drugs in early stages of diabetic retinopathy: results of the EUROCONDOR clinical trial. Diabetes. (2019) 68:457–63. doi: 10.2337/db18-0682 30389750

[52] Jung KI Kim JH Han JS Park CK . Exploring neuroprotective effects of topical brimonidine in experimental diabetic retinopathy. In Vivo. (2024) 38:1609–20. doi: 10.21873/invivo.13611 38936912 PMC11215565

[53] Hernandez C Bogdanov P Corraliza L Garcia-Ramirez M Sola-Adell C Arranz JA . Topical administration of GLP-1 receptor agonists prevents retinal neurodegeneration in experimental diabetes. Diabetes. (2016) 65:172–87. doi: 10.2337/db15-0443 26384381

[54] Zhou Z Sun B Huang S Zhu C Bian M . Glycemic variability: adverse clinical outcomes and how to improve it? Cardiovasc Diabetol. (2020) 19:102. doi: 10.1186/s12933-020-01085-6 32622354 PMC7335439

[55] Ling J Ng JKC Chan JCN Chow E . Use of continuous glucose monitoring in the assessment and management of patients with diabetes and chronic kidney disease. Front Endocrinol (Lausanne). (2022) 13:869899. doi: 10.3389/fendo.2022.869899 35528010 PMC9074296

[56] Hanssen NMJ Kraakman MJ Flynn MC Nagareddy PR Schalkwijk CG Murphy AJ . Postprandial glucose spikes, an important contributor to cardiovascular disease in diabetes? Front Cardiovasc Med. (2020) 7:570553. doi: 10.3389/fcvm.2020.570553 33195459 PMC7530333

[57] Lin YK Fisher SJ Pop-Busui R . Hypoglycemia unawareness and autonomic dysfunction in diabetes: lessons learned and roles of diabetes technologies. J Diabetes Investig. (2020) 11:1388–402. doi: 10.1111/jdi.13290 PMC7610104 32403204

